# Neural Mechanisms of Vicarious Reward Processing in Adults with Autism Spectrum Disorder

**DOI:** 10.1155/2020/8014248

**Published:** 2020-03-21

**Authors:** Rachel K. Greene, Cara R. Damiano-Goodwin, Erin Walsh, Joshua Bizzell, Gabriel S. Dichter

**Affiliations:** ^1^Department of Psychology and Neuroscience, University of North Carolina at Chapel Hill, Chapel Hill, NC 27514, USA; ^2^Department of Psychiatry, University of North Carolina at Chapel Hill School of Medicine, Chapel Hill, NC 27514, USA; ^3^Duke-UNC Brain Imaging and Analysis Center, Duke University Medical Center, Durham, NC 15 27705, USA; ^4^Carolina Institute for Developmental Disabilities, University of North Carolina at Chapel Hill School of Medicine, Chapel Hill, NC 27514, USA

## Abstract

Previous studies examining the neural substrates of reward processing in ASD have explored responses to rewards for oneself but not rewards earned for others (i.e., vicarious reward). This omission is notable given that vicarious reward processing is a critical component of creating and maintaining social relationships. The current study examined the neural mechanisms of vicarious reward processing in 15 adults with ASD and 15 age- and gender-matched typically developing controls. Individuals with ASD demonstrated attenuated activation of reward-related regions during vicarious reward processing. Altered connectivity was also observed in individuals with ASD during reward receipt. These findings of altered neural sensitivity to vicarious reward processing may represent a mechanism that hinders the development of social abilities in ASD.

## 1. Introduction

Autism spectrum disorder (ASD) is a neurodevelopmental disorder characterized by social communicative impairments, as well as rigid, repetitive behaviors and restricted interests [1]. Evidence for impaired social motivation in ASD [2–4], along with enhanced motivation to engage in activities related to repetitive behaviors and restricted interests [5, 6] suggests that the processing of rewards may be broadly dysregulated in ASD. Indeed, recent behavioral and fMRI studies in ASD support this pattern of dysregulated reward processing [7–13].

Although previous ASD studies provide evidence for impaired responses to rewards earned for oneself, few have examined responses to rewards earned for others (i.e., vicarious reward). Here, vicarious reward is defined as an individual's experience of another person's anticipated or consumed reward [14], and the examination of this construct has facilitated a better understanding of the mechanisms behind certain prosocial behaviors [15, 16], as well as social decision-making and learning [17]. There is support for differences in vicarious reward responses in individuals with ASD using behavioral tasks. For example, whereas individuals with ASD do not give less money than controls in simulated charitable giving tasks [18, 19], they do give significantly less money to charities directly benefitting people (as opposed to the environment, for example) and are less impacted by information regarding people-related charities [19]. Relatedly, individuals with ASD are not as influenced by social context in their willingness to give to others, whereas control populations have a tendency to engage in more prosocial behaviors when observed by others [20–24]. Individuals with ASD appear to be somewhat immune to this effect and are equally generous whether they are observed or not [18]. Furthermore, Mosner et al. [25] reported that individuals with ASD demonstrated unimpaired expended effort for monetary rewards for oneself, but they demonstrated reduced sensitivity to reward magnitude parameters when earning rewards for others. Taken together, these behavioral findings support a hypothesis of altered vicarious reward processing in ASD.

However, to date there have been no studies examining the neural correlates of vicarious reward responses in ASD. Previous research in nonclinical populations suggests that giving monetary rewards to others may be experienced as rewarding itself [26] and that similar neurocircuitry that is recruited for the receipt of rewards for oneself may be involved in vicarious reward processing, including the ventral striatum [15, 27–33], dorsal striatum [16, 29], ventral tegmental area [32], insula [16, 29], anterior cingulate gyrus [16, 34, 35], and prefrontal cortical regions, such as orbital frontal cortex (OFC) and ventromedial prefrontal cortex (vmPFC) [16, 28, 32, 33, 36]. Additionally, a meta-analysis by Morelli and colleagues [16] examining vicarious neural reward responses (not restricted to monetary rewards) in typically developing controls (TDCs) found distinct neural activation clusters depending on reward recipient (i.e. self or others). The posterior superior temporal gyrus (STG), the dorsomedial prefrontal cortex (DMPFC), the middle temporal gyrus (MTG), and the superior and middle occipital cortices were more active in response to rewards for others relative to rewards for oneself. Other studies have found preferential activation of the anterior cingulate gyrus in response to vicarious rewards relative to rewards for oneself [37] and revealed significant associations between greater empathic traits and vicarious reward activations in this region [35]. Alternatively, the nucleus accumbens (NAcc), caudate, and thalamus appear to demonstrate greater activation to rewards for oneself relative to vicarious rewards [16].

The present study examined neural activation and connectivity in adults with ASD in response to rewards earned for themselves (standard reward condition) and others (vicarious reward condition) using an adapted version of the monetary incentive delay (MID) task [38]. Given previous findings that individuals with ASD demonstrate reduced sensitivity to behavioral reward magnitude parameters when earning vicarious rewards, but not rewards for oneself [25], the current study hypothesized that group differences in neural activation and connectivity in canonical reward processing regions would be relatively more pronounced in response to vicarious reward anticipation and receipt. Exploratory analyses evaluated relations between neural activation and ASD symptom severity.

## 2. Methods

### 2.1. Participants

This study included 16 right-handed adults with a diagnosis of ASD and 15 right-handed age-matched TDCs (see Table 1). Diagnoses of ASD were supported by the Autism Diagnostic Observation Schedule–Generic (ADOS-G) [41] conducted by a research-reliable assessor with standard clinical algorithm cutoffs. ASD participants were recruited from the Autism Subject Registry maintained by the University of North Carolina at Chapel Hill (UNC-CH) Carolina Institute for Developmental Disabilities (CIDD). Control participants were recruited from a database of TDC participants maintained at the Duke-UNC Brain Imaging and Analysis Center. Acceptance into this control database required participants to score below an 11 on the Beck Depression Inventory [42] and below an eight on the Beck Anxiety Inventory [43]. Informed consent was obtained from all individual participants included in the study.

All participants were male to limit heterogeneity and because gender is related to differences in reward circuitry activation [44]. Five individuals in the ASD group were taking psychotropic medications, including Risperdal, Citalopram, Effexor, Adderall, and Bupropion. Participants were not formally assessed or excluded for co-occurring psychiatric conditions. Because approximately 70% of individuals with ASD also have at least one co-occurring psychiatric condition [45, 46], excluding on the basis of psychiatric comorbidity would significantly reduce the generalizability of the findings to the broader ASD population. Exclusionary criteria for the ASD group included a history of medical conditions associated with ASD, such as Fragile X syndrome, tuberous sclerosis, neurofibromatosis, phenylketonuria, epilepsy and gross brain injury, and severe sensory or motor impairments. Participants had no MRI contraindications and were required to meet a full-scale intelligence (IQ) estimate cutoff of 80. Individuals in the ASD group were administered the Wechsler Abbreviated Scale of Intelligence [39], whereas TDC individuals completed the National Adult Reading Test-Revised (NART-R) to estimate IQ [40]. There were no significant group differences in performance, verbal, or full-scale IQ (see Table 1). Self-reported social communication and interaction impairment was assessed using the Social Responsiveness Scale (SRS) [47].

One participant was excluded because of technical issues related to their high-resolution MRI anatomical image. Therefore, the final ASD group (*n* = 15) included 12 Caucasian participants and three African American participants. The TDC group (*n* = 15) included 13 participants of Caucasian descent, one African American participant, and one Asian participant. Analyses of the standard reward condition (i.e., main effect of standard reward and reward condition interaction analyses) included only 14 individuals with ASD because one participant's behavioral data for the standard reward condition was corrupted.

### 2.2. fMRI Task

The MID task variants used in the current study were adapted from a task originally designed by Knutson and colleagues [38]. Participants completed four versions of this task with four different stimulus types, only two of which are presented here (i.e., reward for self and reward for others). Runs were presented in a randomized order that was counterbalanced across participants. One run involved the opportunity for participants to gain monetary reward ($1 per trial) for themselves if they pressed a button quickly enough following the presentation of a bullseye image (“self” condition). In the other run, participants were informed that they had the opportunity to win money for another participant in the study if they pressed the button quickly enough in response to the bullseye (“other” condition). Participants were informed that the person for whom they were earning money would later participate in the study and that a previous participant had already won money for them. They were not provided with any additional information about the participant for whom they were winning money or how much the previous participant had won for them. The vicarious condition stimuli were identical to the standard condition except for the instructions provided at the start of the run. All instructions were thoroughly explained to participants before the scan session using both verbal and visual instructions.

Each trial of the MID task consisted of: (1) a 2000 ms cue indicating whether monetary reward could be won (a triangle) or not (a circle) on a given trial; (2) a 2000–2500 ms crosshair fixation; (3) a target bullseye presented for up to 500 ms that required a speeded button press; (4) 3000 ms of feedback to indicate whether participants were successful in providing a sufficiently fast response; and (5) a variable length intertrial interval (ITI) crosshair resulting in a total trial duration of 12 sec. For trials in which a monetary reward was possible (reward trials), a sufficiently fast response resulted in the presentation of an image representing a gain of $1 per successful trial, while a slower response resulted in presentation of an “*X*” indicating that no money had been won. For trials in which monetary reward was not possible (nonreward trials), participants were instructed to still respond as quickly as possible to the bullseye image. In these nonreward trials, a sufficiently fast response resulted in presentation of checkmark symbol indicating a successful response and no monetary gain, while a slower response resulted in the presentation of an “*X*” indicating an unsuccessful response and no monetary gain. Potential reward and nonreward trials were aperiodic and pseudorandomly ordered. Each run included 40 trials (50% reward trials, 50% nonreward trials). Participants were instructed to win as much money as possible for themselves or for others and that rewards were contingent on response times. The response time threshold for successful trials was adapted to individual differences in response times, such that all participants were successful on approximately two-thirds of trials (i.e., ∼66.67% accuracy). All stimuli were presented using E-Prime presentation software v. 1.1 (Psychology Software Tools Inc., Pittsburgh, PA, USA) and were viewed through magnet-compatible goggles (Resonance Technology Inc., Northridge CA, USA).

### 2.3. fMRI Acquisition and Preprocessing

Scanning was performed on a General Electric Health Technologies, 3 Tesla Signa Excite HD scanner system with 40-mT/m gradients at 150 T/m/s slew rate (General Electric, Waukesha, WI, USA). Head movement was restricted using foam cushions. An eight-channel head coil was used for parallel imaging. Thirty high-resolution images were acquired using a 3D fast SPGR BRAVO pulse sequence (TR = 7.584 ms; TE = 2.936 ms; FOV = 256^2^ mm; voxel size = 1 × 1 × 1 mm; flip angle = 12°) and used for coregistration with the functional data. These structural images were aligned in the near axial plane defined by the anterior and posterior commissures. Whole brain functional images consisted of 30 slices parallel to the AC-PC plane using a BOLD-sensitive SENSE spiral pulse sequence, at TR of 2000 ms (TE = 30 ms; FOV = 240^2^ mm voxel size: 3.75 × 3.75 × 4 mm; flip angle = 60°). Runs began with four discarded RF excitations to allow for steady state equilibrium.

### 2.4. Motion Correction

In addition to conducting motion correction using FSL's MCFLIRT (FMRI Expert Analysis Tool) [48, 49], volumes with framewise displacement > 0.9 mm [50] were entered into the general linear model (GLM) model as additional confound variables within first-level analyses using FSL's motion outlier detection program (http://fsl.fmrib.ox.ac.uk/fsl/fslwiki/FSLMotionOutliers). All runs included in the analyses were required to retain >40% of their total volumes following the motion outlier correction. Based on this criterion, all runs were included within the analyses. *T*-tests compared diagnostic groups with respect to motion and found that there was equivalent motion in both groups for both conditions for mean and maximum values along all six axes (i.e., *x*, *y*, *z*, pitch, yaw, and roll); all *p*'s > 0.05.

### 2.5. fMRI Data Analysis

#### 2.5.1. Preprocessing

Functional data were preprocessed using FEAT [49, 51] version 5.0.10 in FSL (FMRIB's Software Library, Oxford University; http://www.fmrib.ox.ac.uk/fsl). Preprocessing for all functional data involved the following steps: (1) brain extraction to remove all nonbrain data [48]; (2) motion correction using MCFLIRT [52]; (3) spatial smoothing using a Gaussian kernel of FWHM 5 mm; (4) FMRIB's Improved Linear Model (FILM) prewhitening; and (5) high-pass filtering [52]. FMRIB's Linear Image Registration Tool (FLIRT) [52, 53] was used to register functional images to each subject's T1-weighted structural images with boundary-based registration (BBR) [54]. These coregistered images were then normalized to a standard stereotaxic Montreal Neurological Institute (MNI) space.

#### 2.5.2. Functional Activation Analyses

For all analyses, anticipation and outcome phases were analyzed separately. Masks were thresholded at 25%, binarized, and then combined into a single mask using fslmaths. Higher-level statistical analyses for within- and between-group analyses were carried out using FLAME 1 (FMRIB's Local Analysis of Mixed Effects) [55, 56]. Additionally, automatic outlier de-weighting was employed within FLAME 1 to reduce the impact of prefrontal signal drop out within a few participant runs. Key anatomical regions within the reward system (i.e., NAcc, caudate, putamen, thalamus, insula, anterior cingulate gyrus, orbitofrontal cortex, medial prefrontal cortex, superior frontal gyrus) [57–59], as well as regions shown to preferentially respond to vicarious rewards (i.e., STG, MTG) [16], were defined *a priori* for small volume correction. For this mask, regions were generated separately for the right and left hemispheres in FSL using the Harvard–Oxford cortical and subcortical structural probabilistic atlases. This mask was then entered within group-level models, in which activation clusters were thresholded at *Z* = 2.58. Supplemental whole-brain analyses were also conducted to examine functional activations during both vicarious and standard reward conditions (see Supplementary Materials). Localizations were based on Harvard-Oxford cortical and subcortical structural probabilistic atlases as implemented in FSLView version 5.0.9, and activations were visualized with MRIcron (https://www.nitrc.org/projects/mricron/).

#### 2.5.3. Symptom Analyses

Symptom analyses examined relations between ASD symptom severity, measured by the SRS [60], and functional activation during vicarious rewards in the ASD and TDC groups separately. These analyses were conducted by extracting percent signal change from group-differentiated functional clusters identified within activation analyses. Correlational analyses were then conducted between these parameter estimates and SRS raw total scores.

#### 2.5.4. Functional Connectivity Analyses

Task-based functional connectivity was analyzed using a generalized psychophysiological interaction (gPPI) approach. Voxel-wise models evaluated whole-brain connectivity with functionally and structurally defined seeds. For each participant, mean fMRI timecourses (i.e., physiological regressors) were extracted from seed regions for each task run using *fslmeants* in FSL, then multiplied by each psychological regressor of interest (i.e., Trial Type: Reward, Nonreward) to form the PPI interaction terms. The gPPI model included physiological and psychological regressors, as well as their interaction terms, to describe the unique effect of these interactions above and beyond the main effect of seed time courses and reward conditions. Significant connections were identified in group-level models using a threshold of *Z* = 2.58.

## 3. Results

### 3.1. Behavioral Results

**Figure 1:**
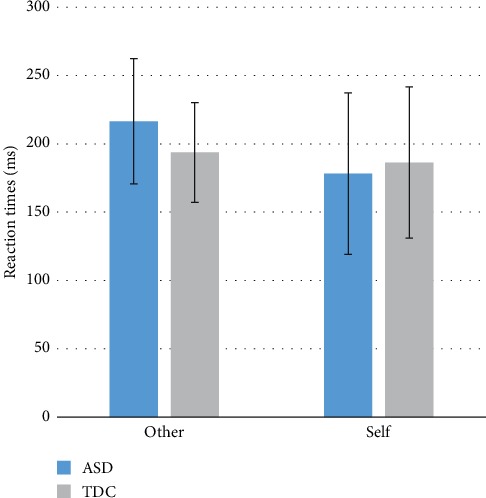
ASD and TDC group-averaged reaction times in response to rewards earned for self and other. The difference between reaction times to personal versus vicarious rewards was only marginally significant (*p*=0.067) across groups. Error bars represent standard deviations of the mean.

Response times (RTs) for successful reward trials are depicted in Figure 1 and were compared via a 2 (Group: ASD, TDC) × 2 (Reward Recipient: Self, Other) mixed ANOVA. These analyses revealed that there was no significant Group × Reward Recipient interaction, *F*(1,27) = 0.79, *p* = 0.38 or main effect of Group, *F*(1,27) = 0.92, *p* = 0.35. The main effect of Reward Recipient neared significance, *F*(1,27) = 3.63, *p* = 0.067, with participants across both groups responding more quickly to reward for Self (*M* = 191.45, SD = 56.44) relative to Other (*M* = 205.50, SD = 42.43). Similarly, accuracy (percent of correct trials) was examined within a 2 (Group: ASD, TDC) × 2 (Reward Recipient: Self, Other) mixed ANOVA. These analyses also revealed no significant Group × Reward Recipient interactions, *F*(1,27) = 0.10, *p* = 0.75, or main effect of Group, *F*(1,27) = 0.00, *p* = 0.99. However, again, the main effect of Reward Recipient trended toward significance, *F*(1,27) = 3.63, *p* = 0.068, such that all participants were slightly more accurate when earing reward for Other (*M* = 0.69, SD = 0.05) relative to Self (*M* = 0.67, SD = 0.05).

### 3.2. Activation Analyses

Activation analyses presented below represent findings from the Reward > Baseline contrast, as there were no significant group differences with respect to the Reward > Nonreward contrast.

#### 3.2.1. Anticipation

Analyses within the TDC group alone revealed significantly greater activation following rewards for Self relative to Other within left orbitofrontal cortex (OFC; see Table 2). However, there were no significant activation clusters with decreased activation in response to rewards for Other compared to Self within the TDC group. Additionally, the ASD group exhibited no differences in neural activation between Self and Other reward conditions. There were also no significant differences in activation between groups for either reward condition main effects or interactions during the anticipation phase.

#### 3.2.2. Outcome

**Figure 2:**
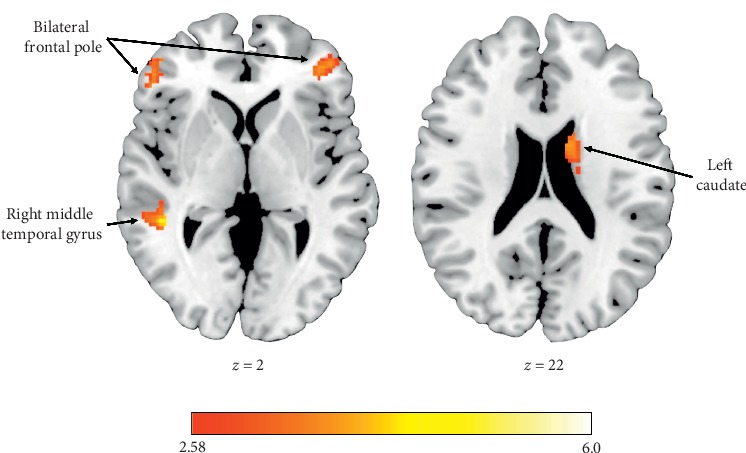
Functional activation clusters showing hypoactivation in individuals with ASD relative to TDC during vicarious reward outcomes.

**Figure 3:**
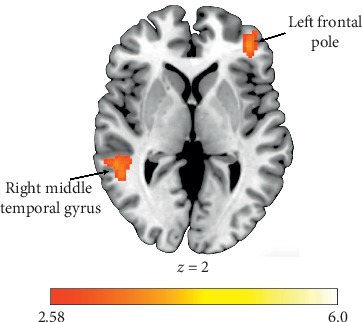
Functional activation clusters showing hypoactivation in individuals with ASD relative to TDCs during vicarious relative to standard reward outcomes.

The TDC group exhibited increased activation within the right middle temporal gyrus (MTG) and the left middle frontal gyrus (MFG) during the receipt of rewards for Other compared to Self (see Table 2). Alternatively, the ASD group demonstrated no significant differences in activation to reward outcomes between recipients. Compared to TDCs, the ASD group showed attenuated responses to rewards for Other within the bilateral frontal pole (FP), right MTG, left superior frontal gyrus (SFG), and left caudate nucleus (see Figure 2). In addition, relative to TDC individuals, those with ASD exhibited significantly decreased activation in right MTG and left FP during reward outcomes for Other relative to Self (see Figure 3).

Main effects analyses for each reward condition by Group are presented within Supplementary Tables [Supplementary-material supplementary-material-1] and [Supplementary-material supplementary-material-1]. These simple effects analyses revealed that both groups showed activation in mesocorticolimbic reward processing regions in response to both reward conditions. Additionally, results from the whole-brain activation analyses are presented in Supplementary Tables [Supplementary-material supplementary-material-1] and [Supplementary-material supplementary-material-1].

### 3.3. Correlations between Functional Activation and ASD Symptoms

ASD symptom severity was not significantly correlated with functional activation during vicarious reward outcomes within group-differentiated clusters identified by the activation analyses described above (i.e., left FP, right MTG, and left caudate nucleus); all *p*'s > 0.05.

### 3.4. Functional Connectivity Analyses

Functional neural connectivity analyses were conducted using a seed constructed from the group-differentiated functional cluster in the MTG identified within the outcome phase activation Group (ASD, TDC) × Reward Recipient (Self, Other) interaction analyses. Because of our *a priori* interest in the NAcc, connectivity analyses also included structurally defined left and right NAcc seeds. Because group differences in activation were restricted to the outcome phase of the tasks, all functional connectivity analyses were restricted to the outcome phase only to constrain the number of analyses performed and, thereby, limit the potential for Type I errors.

#### 3.4.1. Functional Middle Temporal Gyrus Seed

**Figure 4:**
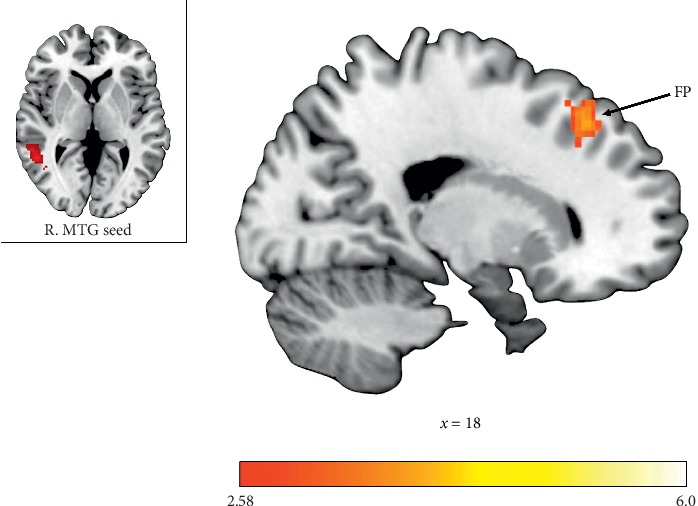
Functional connectivity clusters showing decreased connectivity with right middle temporal gyrus (MTG) in individuals with ASD relative to TDCs during vicarious reward outcomes. FP = frontal pole.

During the receipt of rewards for Self, individuals with ASD exhibited decreased functional connectivity relative to TDCs between the right MTG and the left caudate, right thalamus, right FP, right inferior frontal gyrus (IFG), and left superior parietal lobule (see Table 3). Attenuated functional connectivity was also observed in individuals with ASD relative to TDCs during the receipt of rewards for Others between the right MTG and right FP (see Figure 4). Individuals with ASD showed heightened connectivity, however, between the functional right MTG seed and the left lateral occipital cortex, other regions within the right MTG, and the left lingual gyrus during the receipt of rewards for Others relative to Self.

#### 3.4.2. Structural Right NAcc Seed

During outcomes to rewards in the Self condition, attenuated functional connectivity was observed between right NAcc and the left caudate, right pallidum, bilateral frontal pole, left superior parietal lobule, right thalamus, and left precuneus for individuals with ASD relative to TDCs (see Table 3). Additionally, during reward outcomes for Other, decreased connectivity was exhibited for individuals with ASD relative to TDCs between right NAcc and left caudate, right thalamus, right MTG, left anterior cingulate gyrus (ACG), bilateral frontal pole, and right IFG. No hyperconnectivity with the right NAcc was observed in individuals with ASD relative to TDCs. There were also no significant connections identified in Group (ASD, TDC) × Reward Recipient (Self, Other) interaction analyses with the structural right NAcc seed.

#### 3.4.3. Structural Left NAcc Seed

**Figure 5:**
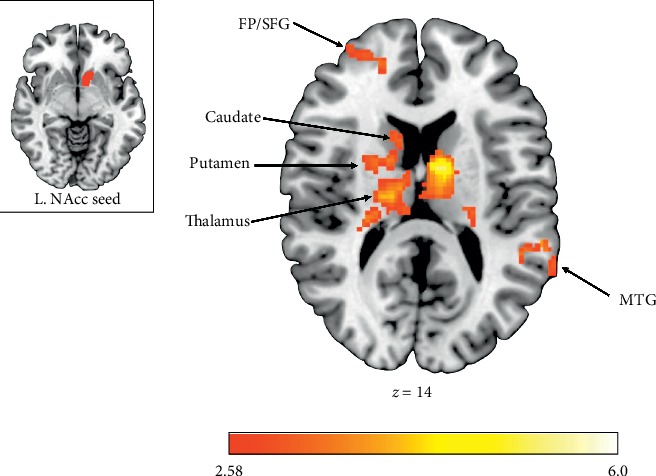
Functional connectivity clusters showing decreased connectivity with the left Nucleus Accumbens (NAcc) in individuals with ASD relative to TDCs during vicarious reward outcomes. FP = frontal pole; SFG = superior frontal gyrus; MTG = middle temporal gyrus.

The left NAcc seed showed similar patterns of hypoconnectivity for individuals with ASD. Specifically, during reward outcomes for Self, individuals with ASD relative to TDCs exhibited attenuated communication between left NAcc and bilateral caudate, left superior parietal lobule, right pallidum, right IFG, left precuneus, and left subcallosal cortex (see Table 3). Connectivity between left NAcc and bilateral caudate, right thalamus, bilateral SFG, bilateral IFG, right FP, left precentral gyrus, left STG, and left MTG was similarly reduced for individuals with ASD relative to TDCs during reward receipt for Other (see Figure 5). Again, there were no brain regions that showed heightened connectivity with left NAcc in individuals with ASD relative to TDC, and there were no significant connections with left NAcc identified within Group (ASD, TDC) × Reward Recipient (Self, Other) interaction analyses.

## 4. Discussion

The goal of the present study was to investigate neural processing of vicarious rewards (“Other” condition) relative to standard rewards (“Self” condition) in adults with ASD. In line with hypotheses, individuals with ASD exhibited attenuated neural activation in key reward regions in response to vicarious reward receipt. Specifically, hypoactivation was observed within bilateral FP, right MTG, left SFG, and left caudate during vicarious reward outcomes in individuals with ASD. Furthermore, right MTG and left FP were preferentially deactivated in individuals with ASD relative to TDCs during vicarious relative to standard reward outcomes. These results corroborate existing ASD reward processing findings that have largely reported a pattern of hypoactivation in response to rewards [61]. The MTG plays a key role in vicarious reward processing [16]; therefore, it is noteworthy that individuals with ASD recruited this region to a lesser extent than their typically developing peers. The MTG has also previously been implicated in theory of mind abilities [62, 63], social perception [64], and empathic judgements [65], and relatively diminished activation within the MTG in individuals with ASD has previously been reported in response to social mentalizing tasks [66, 67]. Taken together, ASD hypoactivation in the MTG may underlie observed deficits in behavioral vicarious reward responses [25], as well as broader social cognitive deficits inherent to the disorder.

Whereas the TDC vicarious reward literature does not specifically implicate striatal regions as being preferentially activated for vicarious reward processing [16], it is noteworthy that individuals with ASD showed reduced activation during vicarious reward outcomes in the caudate nucleus, given its established role in reward processing. The caudate nucleus is a component of the dorsal striatum which plays a key role in the evaluation of action outcomes [68] and assumes the “critic” role within the actor-critic reinforcement model of reward-based learning [69], whereas ventral striatal regions play the “actor” role in controlling and enacting reward-related behaviors [70]. The current findings of ASD hypoactivation within the caudate nucleus during the outcome phase of the vicarious reward condition may, thus, suggest the evaluative role of the caudate nucleus in response to vicarious reward receipt is disrupted in ASD.

Contrary to hypotheses, no significant group differences in activation were observed within the anticipation phase. This indicates ASD-associated differences in vicarious reward processing are constrained to reward receipt, whereas responses to vicarious reward anticipation are unimpaired. Additionally, there were no group differences in response to reward anticipation or outcomes for oneself. This counters studies that have shown differences in neural activation between individuals with ASD and TDCs in response to monetary rewards for oneself [10, 12, 71] but is consistent with others that have found neural responses to standard monetary rewards are unaltered in ASD [9, 13].

To date no study has compared neural functional connectivity of individuals with ASD to TDCs during a reward-specific task. Altered neural connectivity with key reward regions was observed in individuals with ASD relative to TDC for both standard and vicarious reward conditions. The functionally defined right MTG exhibited decreased connectivity with left caudate, right thalamus, right FP, right IFG, and left superior parietal lobule during standard reward outcomes, as well as reduced connectivity with right FP during vicarious reward outcomes. Alternatively, the Group × Reward Recipient interaction results revealed *hyperconnectivity* between the right MTG and left lateral occipital cortex, left lingual gyrus, and other regions within the right MTG. Together, this suggests that, for individuals with ASD, the right MTG may disengage with typical reward-associated regions (i.e., caudate, thalamus, and frontal cortical regions) during vicarious reward processing, while recruiting regions less frequently associated with reward processing. When examining connections with both the right and left NAcc, however, there was a consistent pattern of hypoconnectivity with key reward regions when earning both vicarious and standard rewards. There were no significant interactions of Reward Recipient, suggesting this disconnection of the NAcc is equally as impaired in both vicarious and standard rewards in individuals with ASD. These findings build on the inconsistent literature addressing functional connectivity in ASD. Although they corroborate findings of reduced intrinsic mesocorticolimbic underconnectivity [72], they are in contrast with the resting state reports of frontostriatal hyperconnectivity in ASD [73, 74]. However, comparisons between task-based and intrinsic connectivity results should be interpreted with caution, given inherent methodological differences between the two [75].

Task reaction times showed increased speed of responses to rewards for oneself compared to vicarious rewards, although this effect was only marginally significant. There were no group differences or significant interaction between Group (ASD, TDC) and Reward Recipient (Self, Other) for reaction times. Similarly, individuals across both groups showed marginally greater accuracy when earning vicarious relative to standard rewards, such that responses to rewards for others were typically more accurate than those for oneself. Again, there were no group differences in accuracy and this difference in accuracy based on reward recipient was not moderated by group. Overall, this suggests a group-independent association between increased reaction times and greater accuracy when earning rewards for others relative to earning for oneself.

Examining reward circuitry responses to vicarious rewards is important in light of the established differences in empathy and perspective-taking or “theory of mind” in ASD [76–79]. Notably, empathy is defined as a multidimensional construct, consisting of both cognitive and affective components [80–82]. Individuals with ASD show impairments in the cognitive dimension of empathy, including theory of mind abilities and recognition of emotions in oneself and others, while affective or emotional empathy (i.e., the ability to experience similar emotions as others as a result of the other person's emotional state) appears unimpaired in individuals with ASD [83, 84]. In fact, affective empathy may be heightened for individuals with ASD [85], as evidenced by exaggerated facial expressions in children with ASD in response to the distress of a social partner [86]. Findings from the current study may represent the neural underpinnings of deficits in cognitive empathy and other social cognitive differences in ASD. Additionally, these results may have implications for social learning and decision-making abilities of individuals with ASD. Specifically, reduced sensitivity to vicarious rewards may weaken the effectiveness of social learning strategies like vicarious reinforcement, which asserts that individuals emulate their behavior after seeing others rewarded or praised for certain prosocial behaviors [87]. This may also, consequently, contribute to impaired imitation abilities demonstrated by individuals with ASD [88–90].

By comparing responses to standard and vicarious monetary rewards, this study also addresses existing challenges in investigating social rewards in ASD. Specifically, nearly all ASD reward studies have used monetary rewards as a proxy for nonsocial rewards and faces as a proxy for social rewards. The current paradigm utilizing standard and vicarious rewards provides an avenue to investigate social reward responses in ASD, while controlling for potential confounds related to using a personal monetary reward as a comparison (e.g., the representative nature and visual properties of the stimuli).

Several limitations of the current study should be noted. Although the current study found no significant associations between neural reward activation to reward stimuli and ASD symptom severity, future studies should continue examine this relationship. ASD symptom severity was self-reported by individuals in both groups. The absence of associations between ASD symptoms and neural responses may possibly reflect difficulty reporting on socioemotional states for individuals with ASD [91]. Additionally, further studies with larger sample sizes will be needed to replicate these findings. Although co-occurring psychiatric conditions were not assessed, ASD participants were taking medications that are commonly used to treat psychiatric comorbidities and peripheral behavioral symptoms of ASD (e.g., antidepressants, stimulants, and antipsychotics). Future studies will be needed to see if findings replicate in samples not taking psychotropic agents and formally screened for psychiatric comorbidities. Finally, the implications of this study may be restricted to males with ASD with higher cognitive abilities. Given the significant behavioral [92] and neural [93] sex-based differences in ASD, future vicarious reward processing studies should investigate females with ASD as well.

## 5. Conclusions

In summary, individuals with ASD showed typical neural responses during both the anticipation and receipt of rewards earned for themselves, as well as the anticipation of vicarious rewards. However, individuals with ASD demonstrated relatively diminished activation within reward-related regions during the receipt of vicarious, but not standard, rewards. Altered connectivity with the MTG was observed in individuals with ASD during the receipt of rewards for themselves and others. Additionally, decreased connectivity between the NAcc and other canonical neural reward regions was observed in individuals with ASD during vicarious and standard reward outcomes. These findings of reduced neural sensitivity to vicarious reward receipt may represent a mechanism by which theory of mind abilities and social reward learning are disrupted in ASD.

## Figures and Tables

**Table 1:** Participant characteristics.

	ASD (*N* = 15) Mean (SD)	Control (*N* = 15) Mean (SD)	*t*	*p*
Age	27.97 (10.88)	27.47 (8.60)	−0.14	0.89
Full scale IQ^a^	118.3 (10.51)	117.31 (5.06)	−0.31	0.76
Verbal IQ^a^	115.0 (15.89)	114.62 (5.42)	−0.08	0.94
Performance IQ^a^	117.6 (7.15)	116.13 (5.08)	−0.59	0.56

*Note.* ^*∗*^ *p* < 0.05; ^a^ASD Intelligence Quotient (IQ) scores were calculated based on the Wechsler Abbreviated Scale of Intelligence [39], and TDC IQ estimates were measured using the National Adult Reading Test-Revised (NART-R) [40]. IQ scores/estimates were missing for three TDC participants and one ASD participant.

**Table 2:** Small volume-corrected significant functional activation clusters during rewards for Self and Others.

Phase	Reward Recipient	Region	Hem	*k*	BA	*x*	*y*	*z*	*Z* max
Anticipation	TDC								
	Others > Self	Orbitofrontal cortex	L	167	—	−36	40	−18	3.45

Outcome	TDC								
	Others > Self	Middle temporal gyrus	R	219	—	50	−46	0	3.78
		Medial frontal gyrus	L	191	—	−30	20	50	3.67
	ASD > TDC								
	Others < Self	Middle temporal gyrus	R	445	—	52	−52	8	4.11
		Frontal pole	L	165	—	−46	54	−6	3.74
	ASD < TDC								
	Others	Frontal pole	L	271	—	-46	46	−2	4.31
			R	139	46	50	38	6	3.90
		Middle temporal gyrus	R	155	—	44	−40	2	4.63
		Superior frontal gyrus	L	140	6	−22	18	64	3.51
		Caudate nucleus	L	125	—	−10	−4	18	4.19
	Others > Self	Middle temporal gyrus	R	445	—	52	−52	8	4.11
		Frontal pole	L	165	—	−46	54	-6	3.74

*Note.* Analyses were conducted examining the main effect of Group (ASD, TDC) and Reward Recipient (Self, Other) and interactions between the two factors. However, only significant activations are presented within this table. Hem = hemisphere; *k* = cluster size in voxels; BA = Brodmann area; *Z* max = maximum *z*-value.

**Table 3:** Significant functional connections during reward outcomes for Self and Others.

Seed	Reward Recipient	Region	Hem	*k*	BA	*x*	*y*	*z*	*Z* max
Right MTG	ASD > TDC								
	Other > Self	Lateral occipital cortex	L	693	—	−20	−72	50	3.39
		Middle temporal gyrus	R	670	—	54	−36	−2	3.67
		Lingual gyrus	L	623	—	−12	−66	0	3.39
	ASD < TDC								
	Self	Caudate^†^	L	15412	—	−12	0	12	5.53
		Thalamus	R	—	—	8	−22	14	4.75
		Frontal pole	R	—	10	−28	48	20	4.72
		Inferior frontal gyrus	R	—	—	48	30	8	4.66
		Superior parietal lobule	L	1528	—	−22	−50	58	4.85
		Frontal pole	R	415	—	−8	−54	8	4.11
	Other	Frontal pole	R	250	—	14	36	42	3.86

Right NAcc	ASD < TDC								
	Self	Caudate^†^	L	17711	—	−8	0	12	5.15
		Pallidum	R	—	—	28	−12	−2	4.94
		Frontal pole	R	—	—	52	34	−4	4.76
		Superior parietal lobule	L	—	—	−24	−48	58	4.74
		Frontal pole	L	—	—	−28	50	20	4.66
		Thalamus	R	—	—	8	−24	14	4.65
		Precuneus	L	516	—	−8	−54	8	4.19
	Other	Caudate^†^	L	1948	—	−10	−2	12	5.24
		Thalamus	R	—	—	8	−24	12	4.37
		Middle temporal gyrus	R	—	—	54	−26	−12	4.31
		Anterior cingulate gyrus	L	1098	24	−10	18	32	4.52
		Frontal pole	R	707	—	2	62	34	3.94
			L	345	—	−40	36	0	4.03
			L	230	10	−28	52	18	4.25
		Inferior frontal gyrus	R	402	—	32	−4	52	3.92

Left NAcc	ASD < TDC								
	Self	Caudate^†^	L	20401	—	−8	0	12	5.4
		Superior parietal lobule	L	—	—	−22	−50	58	5.03
		Pallidum	R	—	—	28	−18	−2	4.79
		Inferior frontal gyrus	R	—	—	54	12	8	4.72
		Caudate	R	—	—	12	14	12	4.66
		Precuneus	L	663	—	−8	−54	8	4.33
		Subcallosal cortex	L	261	—	−8	8	−20	4.28
	Other	Caudate^†^	L	1231	—	−10	0	12	5.38
		Thalamus	R	—	—	10	−24	12	4.27
		Caudate	R	—	—	12	12	18	3.68
		Superior frontal gyrus	L	789	8	−22	32	50	4.29
			R	452	—	18	32	50	3.75
		Inferior frontal gyrus	L	430	—	−40	34	0	4.3
			R	408	—	52	30	−4	4.05
		Frontal pole	R	341	—	34	58	16	3.54
		Precentral gyrus	L	307	—	−26	−22	46	3.74
		Superior temporal gyrus	L	292	—	−48	−40	10	3.72
		Middle temporal gyrus	L	230	—	54	−26	−10	4.11

*Note.* Analyses were conducted examining the main effect of Group (ASD, TDC) and Reward Recipient (Self, Other) and interactions between the two factors. However, only significant activations are presented within this table.^†^Peaks are listed first for each cluster with subpeaks listed in subsequent indented rows. NAcc = nucleus accumbens; MTG = middle temporal gyrus; Hem = hemisphere; *k* = cluster size in voxels; BA = Brodmann area; *Z* max = maximum *z*-value.

## Data Availability

The fMRI data used to support the findings of this study have not been made available.

## Supplementary Materials

Supplementary Materials Main effect results of reward recipient on small volume-corrected neural activation in ASD and TDC groups separately and whole-brain analysis results of neural activation during vicarious and standard rewards in ASD and TDC.
