# Supplementary material for: Neural Mechanisms of Vicarious Reward Processing in Adults with Autism Spectrum Disorder
Source: Autism Res Treat. 2020 Mar 21;2020:8014248. doi: 10.1155/2020/8014248 (PMC7115154; doi:10.1155/2020/8014248)
Supplement: Supplementary Materials — Main effect results of reward recipient on small volume-corrected neural activation in ASD and TDC groups separately and whole-brain analysis results of neural activation during vicarious and standard rewards in ASD and TDC. [file 8014248.f1.docx]

**Supplementary Materials**

**Main effect results of reward recipient on small volume corrected neural activation in ASD and TDC groups separately**

Activation analyses described below were conducted using the same small volume correction mask as described within the manuscript.

**Anticipation.** Analyses of functional activation of individuals with ASD during the anticipation of rewards for Self revealed significant activations within right putamen, bilateral MTG, bilateral MFG, left paracingulate gyrus, right precentral gyrus and right SFG (see Supplementary Table 1). In response to rewards for Other, individuals with ASD showed heightened activation in right pallidum, left ACG, left caudate, bilateral thalamus, right MFG, left SFG, right central operculum cortex, and right MTG. Individuals within the TDC group exhibited significant activation during reward anticipation for Self within right putamen, right ACG, right thalamus, bilateral SFG, right MTG, and left frontal operculum cortex. Regarding TDC responses to rewards for Other, analyses revealed augmented activation within right pallidum, left ACG, left caudate, right thalamus, bilateral MTG, bilateral SFG, left frontal operculum cortex, bilateral frontal pole, and right posterior STG.

**Outcome.** Analyses of functional activation of individuals with ASD during the outcome of rewards for Self revealed significant activations within right thalamus, right ACG, right FP, right insular cortex, left MFG, right precentral gyrus, and right MTG (see Supplementary Table 2). In response to rewards for Other, individuals with ASD showed heightened activation in right thalamus, right ACG, right insular cortex, right MFG, left central operculum cortex, and right SFG. Individuals within the TDC group exhibited significant activation during reward receipt for Self right thalamus, right ACG, right precentral gyrus, left FP, bilateral SFG, and bilateral MTG. Regarding TDC responses to rewards for Other, analyses revealed increased activation within left thalamus, bilateral FP, left ACG, bilateral MTG, and bilateral SFG.

**Whole-brain analysis results of neural activation during vicarious and standard rewards in ASD and TDC**

Activation analyses described below were conducted using a whole-brain approach, and, thus, were not confined to specific pre-defined anatomical regions.

**Anticipation**. Whole brain functional activation during anticipation of rewards for Self revealed increased neural activations within right lateral occipital cortex for individuals with ASD relative to TDCs (see Supplementary Table 3). Individuals with ASD relative to TDCs showed decreased activation in right lateral occipital cortex and left postcentral gyrus during rewards for Other relative to Self. Individuals with ASD showed no significant hyperactivation relative to TDCs in response to rewards for Other. Furthermore, there were no significant activation clusters during the anticipation of rewards for Self or Other for either group within the main effect analyses.

**Outcome.** Whole brain functional activation analyses of Self and Other reward outcomes showed no significant activation clusters for individuals with ASD. TDCs, however, exhibited greater activation during reward receipt for Other relative to Self within right precuneus, right lateral occipital cortex, right MTG, left supramarginal gyrus, and left MFG (see Supplementary Table 4). During reward outcomes for Other, individuals with ASD demonstrated relatively decreased activation in right ACG, right MTG, bilateral FP, left SFG, and left parahippocampal gyrus. Interaction analyses revealed that, when compared to TDCs, individuals with ASD showed attenuated activation during reward outcomes for Other relative to Self within right MTG, left IFG, right lateral occipital cortex, right supramarginal gyrus, and left angular gyrus.

Supplementary Table 1

*Small Volume-Corrected Main Effect of Group on Functional Activation During Reward Anticipation for Self and Other*

| **Reward Recipient** | **Region** | **Hem** | **k** | **BA** | **x** | **y** | **z** | **Z max** |  |
| --- | --- | --- | --- | --- | --- | --- | --- | --- | --- |
| **ASD** | | | | | | | | | |
| Self | Putamen^†^ | R | 2986 | -- | 22 | 8 | 2 | 5.27 |  |
|  | Putamen | L | -- | -- | -18 | 6 | 0 | 5.01 |  |
|  | Thalamus | R | -- | -- | 8 | -28 | 0 | 4.98 |  |
|  | Caudate | R | -- | -- | 14 | 14 | 2 | 4.89 |  |
|  | Middle Temporal Gyrus | R | 382 | -- | 50 | -64 | -2 | 3.87 |  |
|  | Middle Frontal Gyrus | R | 371 | -- | 42 | 22 | 24 | 4.08 |  |
|  |  | L | 224 | -- | -48 | 30 | 28 | 3.40 |  |
|  | Paracingulate Gyrus | L | 292 | -- | -8 | 16 | 38 | 4.43 |  |
|  | Precentral Gyrus | R | 174 | 6 | 44 | 2 | 48 | 4.58 |  |
|  | Middle Temporal Gyrus | L | 168 | -- | -46 | -54 | 2 | 3.67 |  |
|  | Superior Frontal Gyrus | R | 159 | -- | 12 | 4 | 64 | 4.54 |  |
| Other | Pallidum | R | 470 | -- | 20 | 4 | 2 | 4.35 |  |
|  | Anterior Cingulate Gyrus | L | 465 | -- | -8 | 12 | 36 | 4.35 |  |
|  | Caudate | L | 447 | -- | -12 | 10 | 0 | 4.57 |  |
|  | Thalamus | L | 384 | -- | -4 | -24 | -2 | 4.00 |  |
|  |  | R | 194 | -- | 8 | -24 | -2 | 3.80 |  |
|  | Middle Frontal Gyrus | R | 296 | -- | 32 | 36 | 34 | 4.05 |  |
|  | Superior Frontal Gyrus | L | 213 | -- | -8 | -10 | 74 | 4.13 |  |
|  | Central Opercular Cortex | R | 163 | -- | 40 | 10 | 6 | 4.08 |  |
|  | Middle Temporal Gyrus | R | 147 | -- | 52 | -42 | 10 | 4.05 |  |
| **TDC** | | | | | | | | | |
| Self | Putamen^†^ | R | 1748 | -- | 20 | 18 | 0 | 5.86 |  |
|  | Frontal Operculum Cortex | R | -- | -- | 34 | 20 | 10 | 4.49 |  |
|  | Caudate | L | -- | -- | -18 | 10 | 12 | 4.36 |  |
|  | Anterior Cingulate Gyrus | R | 770 | 24 | 8 | 6 | 40 | 5.4 |  |
|  | Thalamus | R | 651 | -- | 2 | -28 | 0 | 5.23 |  |
|  | Superior Frontal Gyrus | L | 445 | -- | -10 | -2 | 72 | 4.13 |  |
|  |  | R | 367 | -- | 14 | 6 | 64 | 4.59 |  |
|  | Middle Temporal Gyrus | R | 305 | -- | 42 | -52 | 8 | 4.71 |  |
|  | Frontal Operculum Cortex | L | 213 | -- | -30 | 20 | 14 | 4.72 |  |
| Other | Pallidum^†^ | R | 1271 | -- | 20 | 2 | 4 | 5.16 |  |
|  | Orbitofrontal Cortex | R | -- | -- | 30 | 28 | 4 | 4.91 |  |
|  | Frontal Operculum Cortex | R | -- | -- | 34 | 22 | 10 | 4.82 |  |
|  | Insular Cortex | R | -- | -- | 32 | 16 | 8 | 4.73 |  |
|  | Putamen | R | -- | -- | 22 | 14 | 4 | 4.46 |  |
|  | Anterior Cingulate Gyrus^†^ | L | 1125 | -- | -6 | 16 | 34 | 6.26 |  |
|  | Paracingulate Gyrus | R | -- | 32 | 6 | 18 | 38 | 5.22 |  |
|  | Caudate^†^ | L | 1070 | -- | -16 | 16 | 8 | 4.84 |  |
|  | Putamen | L | -- | -- | -20 | 4 | 10 | 4.83 |  |
|  | Subcallosal Cortex | L | -- | -- | -4 | 16 | -16 | 4.04 |  |
|  | Thalamus | R | 1011 | -- | 12 | -20 | -4 | 5.43 |  |
|  | Middle Temporal Gyrus | R | 815 | -- | 42 | -60 | 6 | 4.86 |  |
|  |  | L | 385 | -- | -48 | -66 | -6 | 4.39 |  |
|  | Superior Frontal Gyrus | L | 594 | -- | -16 | 4 | 66 | 5.28 |  |
|  |  | R | 516 | 6 | 10 | -4 | 70 | 4.62 |  |
|  | Frontal Operculum Cortex | L | 273 | -- | -28 | 22 | 12 | 5.37 |  |
|  | Frontal Pole | R | 223 | -- | 38 | 44 | 28 | 3.44 |  |
|  |  | L | 208 | -- | -24 | 46 | -12 | 3.99 |  |
|  |  | L | 199 | -- | -38 | 38 | 22 | 3.26 |  |
|  | Posterior Superior Temporal Gyrus | R | 154 | 22 | 48 | -24 | -4 | 4.27 |  |

*Note*. Analyses were conducted examining the main effect of Group (ASD, TDC) and Reward Recipient (Self, Other). Only significant activations are presented within this table. ^†^ = Peaks are listed first for each cluster with subpeaks listed in subsequent indented rows. BA=Brodmann Area; Z max=maximum z-value.

Supplementary Table 2

*Small Volume-Corrected Main Effect of Group on Functional Activation Reward Outcomes for Self and Other*

| **Reward Recipient** | **Region** | **Hem** | **k** | **BA** | **x** | **y** | **z** | **Z max** |
| --- | --- | --- | --- | --- | --- | --- | --- | --- |
| **ASD** | | | | | | | | |
| Self | Thalamus^†^ | R | 3799 | -- | 6 | -4 | 12 | 6.88 |
|  | Caudate | R | -- | -- | 12 | 4 | 14 | 5.51 |
|  |  | L | -- | -- | -10 | 0 | 12 | 5.06 |
|  | Insular Cortex | L | -- | -- | -36 | 8 | 6 | 5.37 |
|  | Anterior Cingulate Gyrus^†^ | R | 1144 | -- | 2 | -2 | 44 | 5.64 |
|  | Juxtapositional Lobule Cortex | R | -- | -- | 2 | 0 | 48 | 4.90 |
|  | Anterior Cingulate Gyrus | L | -- | -- | -8 | -2 | 42 | 4.58 |
|  | Paracingulate Gyrus | R | -- | 32 | 4 | 10 | 44 | 4.46 |
|  |  | L | -- | -- | 0 | 12 | 42 | 4.32 |
|  | Frontal Pole | R | 720 | -- | 38 | 40 | 22 | 4.06 |
|  | Insular Cortex | R | 680 | -- | 38 | 6 | -2 | 4.76 |
|  | Middle Frontal Gyrus | L | 440 | 9 | -34 | 36 | 30 | 4.48 |
|  | Precentral Gyrus | R | 436 | 6 | 48 | 6 | 38 | 5.06 |
|  | Middle Temporal Gyrus | R | 273 | -- | 44 | -62 | 12 | 3.81 |
| Other | Thalamus^†^ | R | 2594 | -- | 22 | -28 | 0 | 5.18 |
|  | Thalamus | L | -- | -- | -22 | -30 | -2 | 5.14 |
|  | Anterior Cingulate Gyrus^†^ | R | 1219 | 32 | 4 | 14 | 38 | 5.46 |
|  | Anterior Cingulate Gyrus | R | -- | 24 | 4 | 0 | 46 | 5.37 |
|  |  | L | -- | 24 | -2 | 12 | 32 | 5.34 |
|  | Insular Cortex | R | 522 | -- | 32 | 18 | 4 | 4.35 |
|  | Middle Frontal Gyrus | R | 360 | 8 | 38 | 36 | 44 | 4.03 |
|  |  | R | 269 | -- | 54 | 16 | 34 | 4.02 |
|  | Central Opercular Cortex | L | 268 | 13 | -42 | 0 | 10 | 3.06 |
|  | Superior Frontal Gyrus | R | 165 | -- | 8 | -2 | 70 | 4.88 |
| **TDC** | | | | | | | | |
| Self | Thalamus^†^ | R | 6950 | -- | 6 | -4 | 12 | 7.31 |
|  | Insular Cortex | L | -- | -- | -36 | 8 | 6 | 6.15 |
|  |  | R | -- | -- | 32 | 16 | 8 | 5.98 |
|  | Caudate | R | -- | -- | 12 | 6 | 14 | 6.08 |
|  | Thalamus | L | -- | -- | -16 | -32 | -4 | 5.99 |
|  | Anterior Cingulate Gyrus^†^ | R | 1882 | -- | 2 | 0 | 44 | 6.19 |
|  | Juxtapositional Lobule Cortex | R | -- | 24 | 4 | 6 | 46 | 5.49 |
|  | Precentral Gyrus^†^ | R | 1111 | 6 | 46 | 6 | 38 | 6.65 |
|  | Frontal Pole | R | -- | -- | 38 | 40 | 18 | 4.87 |
|  |  | R | -- | 46 | 50 | 36 | 6 | 3.79 |
|  | Middle Frontal Gyrus | R | -- | -- | 42 | 12 | 30 | 4.46 |
|  | Frontal Pole | L | 774 | -- | -30 | 46 | 30 | 4.59 |
|  | Superior Frontal Gyrus | R | 608 | -- | 12 | 10 | 64 | 5.18 |
|  |  | L | 299 | -- | -16 | -10 | 72 | 5.01 |
|  | Middle Temporal Gyrus | L | 282 | -- | -56 | -64 | 8 | 4.59 |
|  |  | R | 169 | -- | 52 | -56 | 0 | 4.11 |
| Other | Thalamus^†^ | L | 7887 | -- | -22 | -30 | -2 | 6.77 |
|  | Thalamus | R | -- | -- | 6 | -8 | 14 | 6.58 |
|  | Caudate | L | -- | -- | -12 | -4 | 18 | 6.40 |
|  | Frontal Pole^†^ | R | 2778 | -- | 32 | 54 | 24 | 6.00 |
|  | Middle Frontal Gyrus | R | -- | 6 | 46 | 6 | 40 | 5.39 |
|  | Frontal Pole | R | -- | 46 | 50 | 44 | 4 | 5.00 |
|  | Anterior Cingulate Gyrus^†^ | L | 2340 | -- | 0 | 8 | 34 | 7.49 |
|  | Anterior Cingulate Gyrus | R | -- | -- | 4 | 14 | 36 | 6.29 |
|  |  | L | -- | 24 | -4 | -2 | 46 | 5.70 |
|  |  | R | -- | 24 | 4 | 6 | 44 | 5.44 |
|  | Paracingulate Gyrus | R | -- | 32 | 6 | 28 | 36 | 5.47 |
|  | Frontal Pole^†^ | L | 1308 | -- | -30 | 52 | 24 | 4.94 |
|  | Middle Frontal Gyrus | L | -- | -- | -38 | 34 | 20 | 4.56 |
|  | Middle Temporal Gyrus^†^ | R | 1057 | -- | 44 | -40 | 2 | 5.64 |
|  | Superior Temporal Gyrus | R | -- | -- | 46 | -30 | 0 | 5.61 |
|  |  | R | -- | 21 | 54 | -30 | -2 | 5.14 |
|  | Angular Gyrus | R | -- | 39 | 60 | -52 | 14 | 4.37 |
|  | Middle Temporal Gyrus | L | 492 | -- | -52 | -66 | -8 | 5.01 |
|  | Superior Frontal Gyrus | R | 947 | -- | 12 | 10 | 70 | 4.92 |
|  |  | L | 611 | -- | -14 | -8 | 70 | 4.87 |

*Note*. Analyses were conducted examining the main effect of Group (ASD, TDC) and Reward Recipient (Self, Other). Only significant activations are presented within this table. ^†^ = Peaks are listed first for each cluster with subpeaks listed in subsequent indented rows. Hem=Hemisphere; k=cluster size in voxels; BA=Brodmann Area; Z max=maximum z-value.

Supplementary Table 3

*Whole-Brain Functional Activation During Reward Anticipation for Self and Other*

| **Reward Recipient** | **Region** | **Hem** | **k** | **BA** | **x** | **y** | **z** | **Z max** |  |
| --- | --- | --- | --- | --- | --- | --- | --- | --- | --- |
| **ASD > TDC** | | | | | | | | | |
| Self | Lateral Occipital Cortex | R | 535 | -- | 40 | -78 | 44 | 3.68 |  |
| Other < Self | Lateral Occipital Cortex | R | 344 | -- | 54 | -72 | 46 | 3.73 |  |
|  | Post Central Gyrus | L | 293 | -- | -54 | -14 | 26 | 3.58 |  |
| **ASD < TDC** | | | | | | | | | |
| Other > Self | Lateral Occipital Cortex | R | 344 | -- | 54 | -72 | 46 | 3.73 |  |
|  | Post Central Gyrus | L | 293 | -- | -54 | -14 | 26 | 3.58 |  |

*Note*. Analyses were conducted examining the main effect of Group (ASD, TDC) and Reward Recipient (Self, Other) and interactions between the two factors. However, only significant activations are presented within this table. Hem=Hemisphere; k=cluster size in voxels; BA=Brodmann Area; Z max=maximum z-value.

Supplementary Table 4

*Whole-Brain Functional Activation During Reward Outcomes for Self and Other*

| **Reward Recipient** | **Region** | **Hem** | **k** | **BA** | **x** | **y** | **z** | **Z max** |
| --- | --- | --- | --- | --- | --- | --- | --- | --- |
| **TDC** | | | | | | | | |
| Other > Self | Precuneus | R | 429 | -- | 2 | -64 | 34 | 3.74 |
|  | Lateral Occipital Cortex | R | 372 | -- | 54 | -60 | 32 | 3.63 |
|  | Middle Temporal Gyrus | R | 293 | -- | 48 | -46 | 0 | 3.82 |
|  | Supramarginal Gyrus | L | 258 | 40 | -46 | -48 | 56 | 3.71 |
|  | Middle Frontal Gyrus | L | 206 | -- | -30 | 20 | 50 | 3.67 |
| **ASD > TDC** | | | | | | | | |
| Other < Self | Middle Temporal Gyrus | R | 533 | -- | 52 | -52 | 8 | 4.11 |
|  | Inferior Frontal Gyrus | L | 434 | -- | -36 | 30 | 16 | 3.97 |
|  | Lateral Occipital Cortex | R | 380 | -- | 42 | -68 | 14 | 3.91 |
|  | Supramarginal Gyrus | R | 362 | 40 | 42 | -44 | 48 | 3.59 |
|  | Angular Gyrus | L | 239 | -- | -34 | -60 | 18 | 3.43 |
| **ASD < TDC** | | | | | | | | |
| Other | Anterior Cingulate Gyrus | R | 1254 | -- | 12 | 10 | 24 | 4.34 |
|  | Middle Temporal Gyrus | R | 723 | -- | 44 | -40 | 2 | 4.63 |
|  | Frontal Pole | L | 434 | -- | -46 | 46 | -2 | 4.31 |
|  |  | R | 239 | 46 | 50 | 38 | 6 | 3.9 |
|  | Superior Frontal Gyrus | L | 203 | -- | -26 | 18 | 66 | 3.89 |
|  | Parahippocampal Gyrus | L | 178 | -- | -30 | -24 | -22 | 3.76 |
| Other > Self | Middle Temporal Gyrus | R | 533 | -- | 52 | -52 | 8 | 4.11 |
|  | Inferior Frontal Gyrus | L | 434 | -- | -36 | 30 | 16 | 3.97 |
|  | Lateral Occipital Cortex | R | 380 | -- | 42 | -68 | 14 | 3.91 |
|  | Supramarginal Gyrus | R | 362 | 40 | 42 | -44 | 48 | 3.59 |
|  | Angular Gyrus | L | 239 | -- | -34 | -60 | 18 | 3.43 |

*Note.* Analyses were conducted examining the main effect of Group (ASD, TDC) and Reward Recipient (Self, Other) and interactions between the two factors. However, only significant activations are presented within this table. Hem=Hemisphere; k=cluster size in voxels; BA=Brodmann Area; Z max=maximum z-value.
